# Medicine, market and communication: ethical considerations in regard to persuasive communication in direct-to-consumer genetic testing services

**DOI:** 10.1186/s12910-018-0292-3

**Published:** 2018-06-05

**Authors:** Manuel Schaper, Silke Schicktanz

**Affiliations:** Department of Medical Ethics and History of Medicine, University Medical Center Göttingen, Humboldtallee 36, 37073 Göttingen, Germany

**Keywords:** Direct-to-consumer genetic testing, Communication ethics, Advertising, Genetic Counselling, Persuasive communication

## Abstract

**Background:**

Commercial genetic testing offered over the internet, known as direct-to-consumer genetic testing (DTC GT), currently is under ethical attack. A common critique aims at the limited validation of the tests as well as the risk of psycho-social stress or adaption of incorrect behavior by users triggered by misleading health information. Here, we examine in detail the specific role of advertising communication of DTC GT companies from a medical ethical perspective. Our argumentative analysis departs from the starting point that DTC GT operates at the intersection of two different contexts: medicine on the one hand and the market on the other. Both fields differ strongly with regard to their standards of communication practices and the underlying normative assumptions regarding autonomy and responsibility.

**Methods:**

Following a short review of the ethical contexts of medical and commercial communication, we provide case examples for persuasive messages of DTC GT websites and briefly analyze their design with a multi-modal approach to illustrate some of their problematic implications.

**Results:**

We observe three main aspects in DTC GT advertising communication: (1) the use of material suggesting medical professional legitimacy as a trust-establishing tool, (2) the suggestion of empowerment as a benefit of using DTC GT services and (3) the narrative of responsibility as a persuasive appeal to a moral self-conception.

**Conclusions:**

While strengthening and respecting the autonomy of a patient is the focus in medical communication, specifically genetic counselling, persuasive communication is the normal mode in marketing of consumer goods, presuming an autonomous, rational, independent consumer. This creates tension in the context of DTC GT regarding the expectation and normative assessment of communication strategies.

Our analysis can even the ground for a better understanding of ethical problems associated with intersections of medical and commercial communication and point to perspectives of analysis of DTC GT advertising.

## Background

“DNA screening for the important moments in life” [1], “Unwrap you. Celebrate your holiday with the gift of knowledge” [2]. These are only two of the latest examples of promising slogans to be found on websites of DTC GT companies. They suggest genetic information as a special present and DNA screening as a means of achieving a better life. Commercially offered genetic tests, so-called direct-to-consumer genetic test (DTC GT), pose a growing and dynamic market in countries with permissive legislation concerning genetic testing [3]. Faster and much cheaper technology, such as high throughput sequencing and growing databanks of hereditary markers, now make it technically possible for more and more companies to offer and advertise tests for a variety of purposes. These tests range from testing for predisposition to common complex diseases, diagnostics of genetic traits for food intolerances and testing for carrier status of rare genetic diseases to paternity and ancestry tests. The business of DTC GT is typically based on marketing via the companies’ websites, where consumers find information and can order test kits to submit a saliva sample for analysis. Test results are then accessible in a password-secured section of the website. Since the website is the main source of information and the virtual location for ordering a test kit and accessing results, we will, in our following analysis, focus on this medium. However, main lines of our argumentation may also apply to traditional forms of advertising for DTC GT.

The current controversy surrounding DTC GT mainly focuses on health-related tests in a narrow sense, i.e. tests for genetic disorders, risk prediction for common complex disease and carrier status [4]. Ancestry or food allergy tests have gained less attention [5]. The spectrum of concerns includes individual possible psychological harm [6], lack of professional counselling [7], lacking data protection and opaque data protection policies [8] and lacking validity and clinical utility of test results [9, 10]. For example, doubts were raised about the validity of tests making risk predictions based on single nucleotide polymorphisms (SNPs). Also it has been problematized that different companies provide different risk estimates for individuals based on the same methods [11, 12]. Some recent empirical studies have shown that risk information obtained via DTC GT has little influence on changes in health-related behavior [11, 13, 14]. Such findings bolster worries about over-interpretation of health risk information but also lessen the hopes associated with preventive measures or life style changes. Furthermore, there is a worry that lay persons misinterpret the provided information [15, 16]. In sum, more research is necessary to elucidate how the results of DTC GT are perceived by users and what effects they have on them [17].

Regarding the communication practices of DTC GT providers, a systematic review by Covolo et al. [18] concluded that companies emphasize positive aspects of their service in terms of individual empowerment, while dangers and disadvantages (genetic discrimination, emotional burdens) are widely neglected. Most of the 16 studies of the review dealing with DTC GT websites and their content were based on content analysis and quality checks of websites on the provided genetic counselling procedures and the descriptions of risks, benefits and limitations of genetic tests. Marketing strategies, they conclude, overemphasize the positive aspects of the product and the role of genes as a cause of disease. The authors further conclude that it would be important for future policy making to better understand the communicative strategies “that explain how communication can be used to manipulate the beliefs and attitudes of consumers”. However, the studies reviewed mainly focused narrowly on the writing/language-based procedure of information transmission (e.g. [19]). Therefore, they conclude that this communication process could be optimized with more comprehensive, more accurate, more balanced and generally more information to counter these deficiencies. Thus, deficits of information in terms of quality, content and comprehensiveness seem to be regarded as a main problem and a leading rationale of criticism. Following this line of argumentation, some studies now suggest to vary presentation modes to improve the understanding and perception of risks [11, 20, 21]. The improvement of communication is here seen in a change of presentation modes of content, presupposing content is accurate and comprehensive. Shaer et al., for example suggest that genetic risk reports should be presented in the form of “interactive bubble charts”, a special visual display mode that accounts for different items simultaneously [21]. While such efforts are generally desirable, they do not account for problems that may arise from the content itself. For example, it is not helpful if content is better understood when the content is actually inaccurate or in some way misleading. In summary, the current debate tends to focus on what is perceived as lacking in DTC GT companies’ communication and policy. To our understanding it hasn’t yet focused on the ethical implications of the persuasive appeals in DTC GT companies’ communication, existing apart from questions around validity and utility of test results. This also includes the aspect of indirect / impersonal one-way online communication.

In this article, we will therefore highlight a frequently addressed aspect that has not been fully explored in recent criticism of DTC GT, namely the role of advertising (i.e. persuasive communication efforts including images) of companies who operate in this field, from an ethical perspective. Our leading research question is: what are main ethical aspects of communicative means and messages of DTC GT companies? We start by problematizing the use of such means in the field of commercialized medical practice as such by referring to a normative standpoint of medical ethics. In doing so, we want to broaden the ethical perspective for analyzing the general effects of such commercialization in genetics and medicine. Furthermore, we provide an example analysis of morally problematic messages induced by commercial advertisements of such tests by analyzing website messages of DTC GT. Finally, we will conclude that simple approaches of content analysis or quality checks of DTC GT websites fall short in capturing the specific quality of the medium and the persuasive appeals of advertising content, and that ethical analysis of DTC GT should be sensitive to those means of persuasion and their implications. In the following, we examine how the form of communication related to DTC GT poses fundamental ethical challenges because of its online *and* commercial character. We want to critically revise the idea that providing more comprehensive and balanced information is the solution to all ethical problems of DTC GT regarding its communication and information aspects, as the problems go beyond these issues. We start with a reflection on economization and commercialization in the health care context, and subsequently on how these phenomena affect communication practice.

### Normative frameworks of communication in medicine and market contexts

In the following, we want to introduce an analytical distinction of communication principles as defined for medicine/health care and for the market. In practice, of course, there is and always has been a tremendous overlap: in many health care systems of the world, medical care is not free of market principles [22]. However, health economists as well as ethicists have pointed out that there is some tension between the market and healthcare [23, 24] as they ideally mark two poles on the practical spectrum. Our focus is mainly on the role of communication in both areas and how there are ethically relevant consequences when the market and medicine overlap.

Within medicine, communication serves patient information. Furthermore, communication can be seen as a trust-building mechanism for a good doctor-patient relationship [25]. In current concepts of shared decision-making it is the crucial means to ensure that facts and values are exchanged and a decision is based on a joint agreement [26]. Overall, communication can be understood as the main condition to transform a traditional, paternalistic form of professional ethics in which the professional reasons and decides on his/her own into a modern version of a relationship based on contractualistic or deliberative assumptions [27]. Today, communication is seen as core part of professional conduct in the area of medicine: from the ancient ideal of doctor-patient confidentiality to the modern concept of informed consent and counselling procedures, medical professional norms and ethical standards value comprehensive, trustful and objective communication as a cornerstone of a good doctor-patient relationship [25].

Concerning the modern principles of medical ethics, communication serves the purpose of supporting the affected person’s autonomous decision-making. Thus, it is supposed to cover all relevant information, especially about meaning, nature and consequences of the procedures. In the case of genetic counselling, additional requirements are in place, since genetic information, especially risk information, can have severe consequences for an individual in terms of personal well-being and life-planning. Here, the additional claim is made that a realistic picture of chances, risks and utility of genetic testing and its results should be drawn [28, 29]. Generally speaking, the communication in this context should provide comprehensive and truthful information in order to put patients or research participants in a position where they can make a well-informed decision (legally defined as informed consent) based on the knowledge of possible consequences of their decision. Further, the aspect of non-directiveness is crucial to genetic counselling which may be historically explained as a compensation reaction to abusive practices in human genetics. It has been suggested that it reflects a wish of medical professionals to distance themselves from their clients’ decisions, especially when it comes to delicate cases of reproductive decision-making [30, 31], and that there is also an economic risk for individual doctors or medical institutions, when they are held responsible for certain consequences of decisions they advised [32]. Non-directiveness thus means that professionals do not provide personal opinions or advice for decision-making as it might be common and expected by patients in many other areas of health care. The appropriateness and factual application of a non-directive approach in genetic counselling is nonetheless a matter of debate and a shared decision-making model may be more suitable. However, the aim of genetic counselling, even though operating with attempts to influence clients in certain situations, would remain in line with their best interest [33]. Particularly, it is this latter aspect of communication that deserves special attention when it comes to DTC GT. Some empirical studies have shown that non-directiveness is not necessarily a reality in actual counselling practice [34] yet it persists as moral ideal that is still paradigmatic in many respective guidelines and recommendations like the UN declaration on human genetic data, the code of ethics of the National Society of Genetic Counselors in the US or the Guidelines of the German Society for Human Genetics (GfH) [28, 35, 36]. Even though this ideal may change with the changing role of genetics in medicine [37] it is important to recognize that it is absent in DTC GT and that in a commercial context there is a clear directive interest. Another important reason why commercial genetic testing deserves special attention is the exceptional nature of genetic data: first, genetic risk information is probabilistic in nature and includes a strong element of uncertainty. The status of such data, in terms of clinical utility, is thus not clear; second, genetic information is not only individual information, as it refers to the genetic make-up also shared by relatives who may be subject to the effects of “knowing” their DNA as well.

On the other hand, communication in the market sphere is characterized by a less distinct ethical framework. However, there are laws and regulations directly or indirectly serving consumer protection in the EU and the US. In the US, DTC genetic tests may be freely advertised if approved by the Federal Food and Drug Administration (FDA) [38]. Apart from varying national regulations, a EU-wide regulation is currently underway. The initial draft intended to ban DTC advertising of genetic tests but the final version includes restrictions on advertising of genetic tests such as a prohibition of false claims about the product’s properties and withholding associated risks [39].

Also, the advertising industry itself has established few ethical guidelines. Lying and cheating is regarded as morally unacceptable and in many circumstances also legally problematic. However, advertisement and the presentation of imbalanced information is regarded as a constitutional part of market communication. Standards do exist that require companies to provide truthful product information in their advertising as well as in the labeling of their products [40]. There is, however, no direct equivalent to the principles of biomedical ethics in commercial communication. Instead, voluntary initiatives of the advertising industry such as the *Advertising Self-Regulatory Council* in the US or the *German Advertising Standards Council* (Deutscher Werberat) are in place. These voluntary commitments of the economy provide guidelines and serve as a means of self-regulation. Yet (at least in Germany) together with laws regulating competition and the competing market players, they form a regulatory structure with a form of *inherent ethics* that is maintained by different stakeholder groups [41]. Thus, commitments of the industry as well as legal restrictions set limits to what is allowed in advertising. The nature of advertising as a type of communication directed at influencing behavior of the targeted individuals, however, is not touched by such efforts. This significantly differs from the ethical framework in medicine where communication directed at patients should comply to the principle of respect for autonomy [42]. Consequently, advertising as a form of communication in medicine immediately becomes ethically problematic, as individuals should not be manipulated in their decision-making.^1^ We therefore assume that the market and medicine are seen as contrary parts on the spectrum of provision of mecical goods. This assumption is further supported by the fact that all countries except for the US and New Zealand have banned advertising of prescription drugs and medical devices [44]. In the case of the US we may assume it to be an expression of a cultural orientation towards individual self-responsibility (not only) for health and a strong tradition of a liberal economy.

Normative frameworks codifying communication therefore exist in both fields. To sum it up, the medical profession should communicate on the basis of its commitment to the autonomy and beneficence of patients [45, 46]. Since genetic risk information can have a severe psychological impact on at-risk individuals the principle of non-maleficence has turned out to be relevant for the communication processes of genetic counselling as well [26]. Our argument points therefore at the tension between persuasive efforts in advertising as common in market communication and the medico-ethical communication ideals.

### Persuasive communication in direct-to-consumer advertising

What are the implications when genetic tests are offered as a consumer’s good on the free market and yet are not or only marginally embedded in the current medical ethical framework? A special focus should be given to the advertisement character and its ethical implications. As explained above, the main principles of the market require that products and services are advertised for the sake of increasing sales numbers and thus serve the main interest of the salesperson. Commercial websites hint at products or services but also present an interactive platform for communication and financial transactions that represents their company. It is therefore important to have a closer look at their content and structure: following common design principles of commercial websites, the key product is usually advertised as the central content on the main page and the purchase interface is just one click away. Supplementary content such as navigation links allow users to navigate to other subpages of the website with additional content and features.

Among various definitions of advertising, one can derive a common understanding that it is intentional communication aimed at behavior changes of the recipients, for the purpose of distributing a product. Based on insights from the field of psychology, the behavioral changes are achieved by changing attitudes or self-images of the consumers and creating desires [47, 48]. We call this its persuasive character, which is based in the utilization of rational and/or arational means [49–51].^2^

The idea of products and services being advertised by providers that compete for consumer attention is a given fact in modern western societies. Advertising is not only a significant sector of business activities but also an area of extensive research in various disciplines and an important cultural phenomenon of modern societies [48, 50]. However, this type of communication raises general concerns with regard to its legitimacy and ethical implications [50, 52–54]. Discussions revolve around the relationship between ends and means of communication, assuming that the means used to achieve certain ends affect the quality and nature of those ends [54].

Advertising operates with a set of seductive qualities and uses techniques from social psychology like priming and evaluative conditioning, methods of influencing attitudes and opinions. Biegler [55, 56] provides an example that shows the use of such techniques in marketing of medical products and how they produce false or inaccurate beliefs. The pairing of information with positive images, for example, lead participants in a study to believe that a drug is “safer, more effective, and more beneficial”, a belief which did not occur in the control group. In another study, Biegler & Vargas examined the effect of images in DTC advertisement for prescription drugs. They state that images can be seen as arational, emotional or even manipulative means, concluding that the used imagery caused viewers to hold beliefs that are explicitly denied in the very same commercial. They identify a bias in US legislation regarding pharmaceuticals toward *“propositional content”*, meaning concrete presentations of facts about a product. In contrast, *“nonpropositional content”*, consistent of images, audio and other content, is being disregarded even though it is an important part of the message. They propose to further examine the medial quality of advertisement to be able to assess its ethical dimension: *“more research and debate are needed to determine the permissibility of this and other forms of nonpropositional persuasion”* [57]. The term ‘nonpropositional content’ seems problematic to the extent to which it literally dismisses the idea that images contain messages, while the conceptional use of the term claims the opposite. The term “implicitly propositional content” may therefore be more suitable to capture the actual meaning of it, maintaining the notion of suggestive character.

The use of subtle persuasion techniques, according to Biegler, undermines patient autonomy and raises doubt whether such advertisement should be restricted. Here lies an important distinction from the concept of nudging. In the medical field, nudging refers to attempts to drive choice in a beneficial direction while preserving autonomy, qualifying this action as a form of libertarian paternalism, an action for the own good of the patient [58].

From an ethical point of view, it is especially problematic that to some degree, the induction of false beliefs may be intended by commercial providers. The occasional and sometimes willful induction of false beliefs can be described as a manipulative action that potentially infringes on the autonomy of recipients. Their own ability to reason and judge independently is being compromised since they do not necessarily become aware of how their attitudes and, consequently, their decision-making are influenced in subtle ways [50, 51, 59]. Thus, it may be expected that advertising uses all means legally available in order to achieve the desired effect, even though ethical permissibility remains questionable, at least in those cases where advertising makes use of emotional appeals not in line with the recipients’ original needs [50].

When we transfer these general considerations to the context of DTC GT we can conclude that websites of DTC GT companies do not provide neutral technical information but contain many messages that classify as advertisement [60]. Websites as a medium are particularly of interest here, as they not only consist of the presentation of text but especially of their visual makeup. This multi-modality adds to the complexity of media messages and requires appropriate methodologies as held, for example, by a social semiotics approach and modern cultural studies [61–63] which we also used for our brief case study (see below). Our analysis, therefore, goes beyond a simplistic content analysis of texts.

It is noteworthy here that another aspect of DTC GT websites is ethically relevant which is related to the medium itself. The communication and handling of information is different in interaction with a commercial website than it is in a genetic counselling process. The face-to-face communication in genetic counselling requires a certain amount of time, effort and attention which ensures, at least to some extent, mutual understanding and a careful decision-making process. The counselor can adapt to the clients’ needs and questions appropriately and the character of the procedure is serious. The online purchase requires no such efforts and consumers are more likely to just rush through the process. Terms and conditions of the service may be perceived as mere obstacles and just ignored and avoided by checking a box. The seductive quality of virtual buttons [64] tempts users to just click through and reach the end of the ordering process. In sum, the entire media context and the content of a commercial website reframes undergoing genetic testing as a quick and easy consumer goods purchase and thus disregards the special qualities and potential implications of such procedures (cf [65]).

## Methods

As we have argued above, DTC GT websites can be understood as a form of advertising that is likely to be using persuasive communication techniques including visual material to influence consumer perceptions and, in consequence, attitudes and behavior. Here, we do not provide a systematic analysis of all or many DTC GT which would exceed the purpose of this more argumentative article. However, following a case study approach [66, 67], we present an example analysis of snippets of three DTC GT companies’ websites to illustrate how both the textual and visual components can be analyzed and thereby underpin our arguments regarding the communicative complexity and the resulting ethical relevance. For this purpose we have selected three different companies from the US, Europe and Asia to reflect the phenomenon on an international level. We take the example websites and snippets as illustrative cases to demonstrate different key issues.

## Results

Overall, we would like to highlight three main critical areas: (1) the use of material suggesting medical professional legitimacy as a trust-establishing tool, (2) the suggestion of empowerment as a benefit of using DTC GT services and (3) the narrative of responsibility as a persuasive appeal to moral self-conception.

**Fig. 1:**
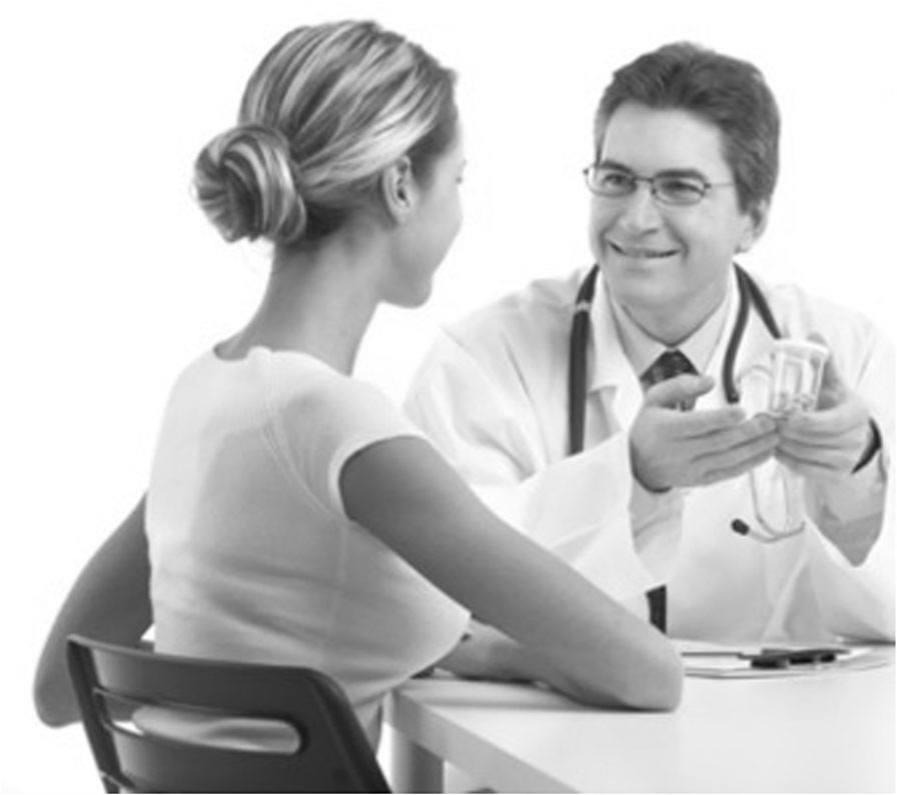
Use of imagery of medical professionals. http://www.geneplanet.com. Image is not included in the creative commons licence for the article and printed under the condition of fair use [94]

For an example of the first aspect see Fig. 1. The use of such material may be misleading in that it suggests involvement of a physician in the process even though, as is the case in this company, the marketing is directed at the consumer and the information of the website does not state that a physician is required to order sample kits or to access the test results online. It may be argued that the image used here is a mere illustration of a situation where a client talks to his/her doctor about his/her health. The contextual use, however, lets the website benefit from the display of a physician in a face-to-face counselling situation. In this situation a woman seen from behind serves as a placeholder for the recipient allowing immersion into the depicted situation. The image thus evokes trust and sympathy which are linked to the content of the website, even though pre-test counselling is not part of the offer.

**Fig. 2:**
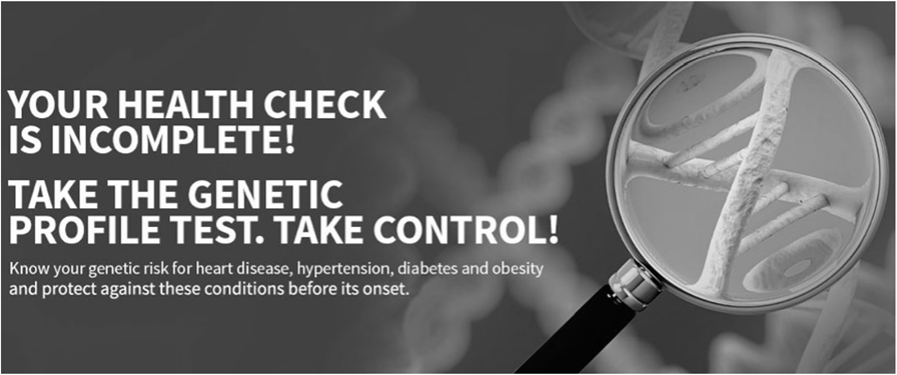
Intimidating message and visual appeal to curiosity and a wish to take action. http://www.xcode.in. Image is not included in the creative commons licence for the article and printed under the condition of fair use [95]

An example for the use of the concept of ‘empowerment’ is provided in Fig. 2. Written in capital letters, the website suggests “YOUR HEALTH CHECK IS INCOMPLETE! TAKE THE GENETIC PROFILE TEST. TAKE CONTROL!” and in smaller font: “Know your genetic risk for heart disease, hypertension, diabetes and obesity and protect against these conditions before its (sic!) onset.”, while the background image of a magnifying glass enhancing the view on a DNA strand symbolically signals that, somehow, important information in the DNA has been overlooked that can now be accessed to complete the health check. Apart from the intimidating message, the text clearly suggests that by undergoing the genetic test, consumers can make up for a lacking health check themselves (social meaning of empowerment) and take control as well as take preventive measures against the diseases mentioned once they know their genetic risk for them (control meaning of empowerment). At the same time, the image appeals to a sense of curiosity and lust for power, a wish to look into detail and become an investigator taking action in uncovering a mystery or looking for traces in a criminal case – someone who is indeed in control. However, undergoing genetic testing is neither a necessary condition to take preventive measures against these particular diseases, nor does knowledge of the risk of getting them equal an increase in control. It remains a fact, however, that the information can be accessed without consultation of a medical professional. Read in this manner, the idea of empowerment is correct, read in the other, it is rather shallow, yet the line between the two is blurred.

A third aspect is responsibilization, i.e. a shift in ascribed responsibility: In 2013, the FDA banned the US website of 23andme from offering genetic testing for predisposition to Alzheimer’s Disease (AD), among other diseases [68]. However, according to information on the website that service is still available outside the US [69]. Although there is increasing critique of giving people access to this particular information, many people say that despite their fear of AD they would rather know their risk than remain in the dark about the danger [70]. For some, knowing their APOE status and AD risk may encourage them to engage in activities to fend off the disease [71] or may prompt them to participate in clinical research that could lead to more information about the causes and possible cures for AD [72]. The latter statement, in particular, must be understood in the context of overall preventive action and risk planning underlying the rhetoric of 23andme “Take a more active role in managing your health. Knowing how your genes may impact your health can help you to plan for the future and personalize your healthcare with your doctor” [73]. A message is mobilized here that appeals to a feeling of responsibility for personal health that goes beyond empowerment in a wide sense, as it implies that until now one has been stuck in a passive role, doing nothing, and now action becomes necessary [74].

**Fig. 3:**
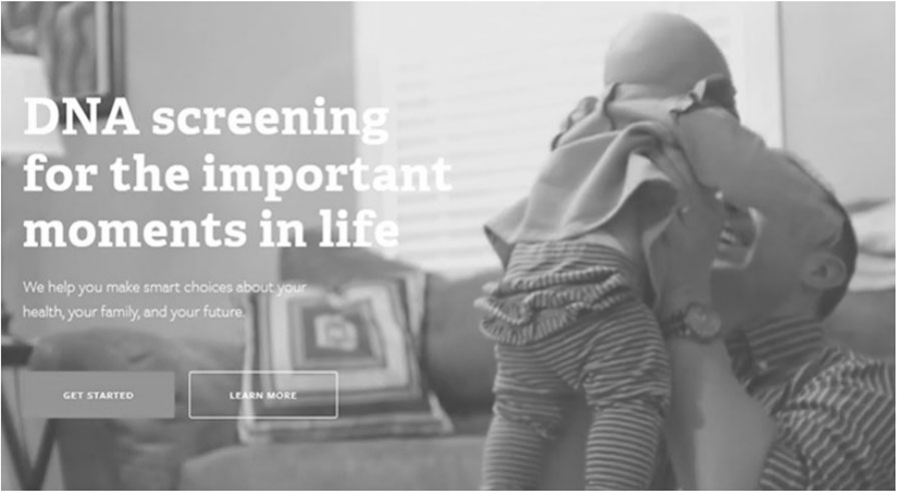
Use of visual material in DTC GT as an appeal to feelings of responsibility. http://www.counsyl.com. Image is not included in the creative commons licence for the article and printed under the condition of fair use [96]

Another example is provided in Fig. 3: An animated graphic displaying fictional cut scenes from key moments of a happy life (a child taking its very first steps with the help of its parents, a woman playing with a dog outside, people celebrating at a family gathering) builds the background for yet another persuasive appeal: “DNA screening for the important moments in life. We help you make smart choices about your health, your family and your future”. Apart from possibly relating to the moral notion of good life here, this message mobilizes a rhetoric of responsible choices and introduces the thought of “smart” decisions affecting not only one’s own personal health but also one’s family’s. By embedding this message in images transporting a sense of community, the importance of close social relationships and the rewards of parenthood, the message calls to a feeling of responsibility towards others. The selling point here is that by using the service a contribution is made to the well-being of close others.

## Discussion

The use of imagery of the type from the first example works as a trust-building device relying on the power of the image of the medical profession. This use of images has also been detected in Borry, Shabani & Howard’s work [75]. According to their analysis, DTC GT websites share analogies to advertising of pharmaceuticals. They argue for the suggestive power of *nonpropositional content* that can also be identified for commercial genetic testing, observing the use of images of doctors in some DTC GT websites even in cases where the marketing model of the provider does not include involvement of a physician. However, only an analysis of the images used can reveal the manner in which they achieve their effect.

The second critical aspect resulting from the use of persuasive communication methods in DTC GT is the utilization of the concept of ‘empowerment’. The meaning of empowerment is indeed twofold: Empowerment entails the ability of individuals or groups to take control of their circumstances and exercise power [76], which, in this case, may be achieved by accessing information without consulting a medical professional and thus being less dependent on an expert. The gate-keeping function of medical professionals is undermined and the individual is put in a situation where he/she is not just treated but also is able to negotiate. In this sense, empowerment means gaining new opportunities for action that were not available before. It represents a shift in power relations in a social context. Understood in this narrow sense, the notion of empowerment is correct, whether the acquired information is helpful or not. This refers to a social meaning of empowerment.

There is, however, an additional notion attached to the term: it covers an alleged increase of control exceeding access to information and overcoming the dependency of medical experts, because it may also imply control over the individual future based on better (health) decisions in the present. In this sense, empowerment refers not only to a power shift in doctor-patient/consumer relationships but to the increase in the capacity to control and create one’s own future health and fate. This notion is an implicit claim not backed by scientific evidence but it clearly has a positive persuasive connotation serving marketing goals, blurring the line between the two meanings of the term. Our observation here is in line with research by Liu and Pearson [77] and Covolo et al. [78] who observe the use of empowerment in DTC GT as a strong emotionally laden selling point. However, the visual means enhancing those emotional appeals were only touched upon slightly in their research.

The phenomenon could also be described in terms of the concept of personal utility: In the second example (Fig. 2) we observe, that the promise of a health benefit exist on a textual level, and the appeal to curiosity and a wish for control are integrated on a pictorial level. The latter corresponds to a merely perceived, personal utility not necessarily based on clinical utility [79].

The appeal to responsibility corresponds with the increasing awareness of aging and the willingness of taking responsibility for life years gained [80]. Such responsibility is sometimes embedded in a naive lay interpretation of genetics, namely to rely on genetic determinism even for common complex diseases. This wish for planning later life is – according to our hypothesis – motivated by moral underpinnings of responsibility towards one’s family and oneself, and to a lesser part towards the society. The narrative of active roles as well as the images of family life and parental care draw our attention towards a new approach, critically discussed as responsibilization [81, 82]. Such responsibilization is, however, problematic if the causalities remain vague or unproven or if the process ends in a social practice of blaming and loss of solidarity [83]. At least for societies with a publically financed healthcare system, this tackles a cultural development corresponding to individualization and self-optimization [84] that needs to be discussed and critically reviewed in future work.

The use of such arational persuasive appeals designed to convince consumers to undergo a genetic test would not be ethically acceptable in face-to-face genetic counselling. This gap reflects the ethical divide between medical ethics and market principles. While the patient’s autonomy is paramount in clinical genetic testing, it becomes a means to an end in commercial genetic testing communication to persuade consumers to develop particular attitudes and show behavior in favor of the DTC GT companies’ economic success. Thus, while genetic counselling per definition does not operate with persuasive communication methods but ideally aims at an open-ended, non-directive process, this claim is not inherently respected in DTC GT commercial practices. The communication aims are in the former medical-consultant while in the latter profit-oriented by nature. The DTC GT companies’ communication is thus ethically problematic when we take into consideration that genetic testing may be a source of harm and may also affect third persons.

We consider two counter-arguments one can raise against this statement. *1. People are used to being confronted with influencing messages, so there is nothing to worry about*. Even though the first part of this statement is factually correct, it would not affect the validity of criticism of the use of persuasive means. On top of that, this argument is a good example of an is-ought problem: just because it is the case that people are constantly confronted with persuasive appeals as consumers this does not mean that this should be the case or that it is not ethically problematic. *2. As Rothschild* [85] *suggests, marketing of public health issues in order to get people to adopt certain behaviors or attitudes may be more effective for public health than education or even the coercive force of law, and may be therefore ethically justified.* However, this is only true if the aim of getting as many people as possible to undergo predictive genetic testing can be clearly, uncontroversially identified as good in itself. This is, however, not the case as the ongoing controversies concerning the clinical and personal utility show, whether for medical or personal purposes. Even if it were, the means by which this end is achieved remain ethically problematic which affects the quality of that end. Research on the use and effects of DTC GT on health behavior has, thus far, not provided clear evidence of health benefits or significant positive changes in health behavior [18, 86, 87]. Contrarily, we have to consider risk compensation as a potential side effect of having personal risk information providing a feeling of safety, leading to deterioration instead of improvement of health behavior [88].

## Conclusions

As we have argued above, DTC GT websites as marketing and advertising platforms contain persuasive messages and arational appeals that pose ethical problems. Apart from the ethical tension between solidarity- or market-oriented health care as such, our analysis points at a more fundamental problem. The question is how the public and patients can be empowered to detect and reflect the complex, persuasive forms of communication that is taking place increasingly in a digitalized market. Persuasive appeals operate on a spectrum [50] and thus pose an ambiguous way of influence in regard to autonomy as they tend to bypass the individual’s capacity to rationally reason and make decisions based on facts. This, however, seems inappropriate, given the critical nature of genetic risk information and its possible impacts that warrant a careful weighing of options. As we have shown, at least some companies rely in their advertising on an appearance of medical legitimacy to gain consumers’ trust, thus utilizing the trustworthy “image” of medicine and doctors for commercial purposes. While there is no clear evidence that DTC GT leads to actual harm in consumers, it remains problematic to address them in a way that is meant to influence their decision-making. The fact that the issue of harm has been investigated repeatedly [18] shows that there is some agreement among scholars that there is at least a risk for harm. We believe this risk should be clearly communicated and not distracted from.

The modes and contents of the advertising messages should therefore be considered in ethical assessments of DTC advertising of genetic testing. We suggest that the actual implications of genetic testing for consumers should be in line with the values and ideas the advertising appeals to, apart from the question whether harm is involved or not. We have further compared the commercial modes and methods of communication with the standards in place for medical genetic counselling and discussed examples of DTC GT website communication that reflect these problems.

We do recognize that there is a variety of reasons why consumers purchase genetic tests and that they may have personal utilities and different values for them that go beyond clinical utility [89]. Nonetheless, the literature on user motivations also shows that improving health is among the most important factors [17, 90–92], and there are also studies that have shown that advertising contents correspond to this motive [93].

Our findings do not allow for a full proposal of recommendations for policy changes in the regulation of DTC advertising of genetic testing, yet we suggest that multimodal analysis of communication content should be considered in ethical examinations of such advertising to gain an in-depth understanding of the explicit and hidden messages of such communication which are both ethically relevant. To gain better understanding of the persuasive appeals in DTC GT, it is necessary to develop a methodological approach that allows us to pay more attention to the details and also visual components to reveal the moral and scientific messages in detail. Adding knowledge about the multimodality of communication allows us to get a more complete picture of the advertising content and make better ethical judgements of DTC GT companies’ advertising. As some companies have seized selling tests directly to consumers and involve doctors as gatekeepers instead, there may be a shift in the ways they address consumers and advertise their services, posing an interesting field of future empirical research.

## Abbreviations

AD: Alzheimer’s disease
APOE: Apolipoprotein E
DNA: Deoxyribonucleic acid
DTC GT: Direct-to-consumer genetic testing
EU: European Union
FDA: Federal Food and Drug Administration
GT: Genetic testing
US: United States

## Footnotes

1 Similar concerns are being discussed in the debate about nudging. While parallels are worth mentioning, the nudging debate revolves around the question whether it is permissible for the state to nudge citizens for their own good [43].

2 Arationality refers here to means that do not influence reasoning by using rational arguments but by evoking emotions or appealing to cognitive heuristics / stereotypes. This does not mean, however, that this type of influence does not follow certain principles or that the way it works does not have its own rationale. Arational means are means that are meant to bypass the reasoning process by making someone feel in a certain way instead of giving them something to think about. For a detailed discussion see Sawicki 2013 [49].
